# Crosstalk between glucagon-like peptide 1 and gut microbiota in metabolic diseases

**DOI:** 10.1128/mbio.02032-23

**Published:** 2023-12-06

**Authors:** Yuan Zeng, Yifan Wu, Qian Zhang, Xinhua Xiao

**Affiliations:** 1 Department of Endocrinology, Key Laboratory of Endocrinology, Ministry of Health, Peking Union Medical College Hospital, Peking Union Medical College, Chinese Academy of Medical Sciences, Beijing, China; Oswaldo Cruz Foundation, Curitiba, Brazil

**Keywords:** glucagon-like peptide 1, gut microbiota, type 2 diabetes, prebiotics, probiotics

## Abstract

Gut microbiota exert influence on gastrointestinal mucosal permeability, bile acid metabolism, short-chain fatty acid synthesis, dietary fiber fermentation, and farnesoid X receptor/Takeda G protein-coupled receptor 5 (TGR5) signal transduction. The incretin glucagon-like peptide 1 (GLP-1) is mainly produced by L cells in the gut and regulates postprandial blood glucose. Changes in gut microbiota composition and function have been observed in obesity and type 2 diabetes (T2D). Meanwhile, the function and rhythm of GLP-1 have also been affected in subjects with obesity or T2D. Therefore, it is necessary to discuss the link between the gut microbiome and GLP-1. In this review, we describe the interaction between GLP-1 and the gut microbiota in metabolic diseases. On the one hand, gut microbiota metabolites stimulate GLP-1 secretion, and gut microbiota affect GLP-1 function and rhythm. On the other hand, the mechanism of action of GLP-1 on gut microbiota involves the inflammatory response. Additionally, we discuss the effects and mechanism of various interventions, such as prebiotics, probiotics, antidiabetic drugs, and bariatric surgery, on the crosstalk between gut microbiota and GLP-1. Finally, we stress that gut microbiota can be used as a target for metabolic diseases, and the clinical application of GLP-1 receptor agonists should be individualized.

## INTRODUCTION

Annual health spending on diabetes imposes a huge burden on society, projected to grow to 845 billion dollars in the United States by 2045, and there are huge differences between countries (1). Therefore, treating type 2 diabetes (T2D) is important. Glucose and weight control are the fundamental steps (2, 3). Recently, changes in gut microbiota composition resulting from diet, drugs, and obesity have been considered one of the pathogeneses of T2D [reviewed in references (4, 5)]. In healthy subjects, oral glucose triggers a stronger insulin secretion response than intravenous glucose because of the secretion of incretin from the gastrointestinal tract, known as the “incretin effect.” Clinically, incretin-based drugs include glucagon-like peptide 1 receptor agonists (GLP-1 RA) (such as liraglutide) and dipeptidyl peptidase 4 inhibitors (DPP-4i) (such as vildagliptin). These drugs are effective in the individualized treatment of T2D with or without obesity and have been used clinically for more than a decade (6–8). In addition, bariatric surgery increased intestinal hormones such as GLP-1 and peptide YY (PYY) (9) and changed the composition of gut microbiota (10) and bile acids (BAs) (11), which all enhanced GLP-1 responses in obese individuals with T2D.

Microbiota refers to a community of microorganisms that colonize a particular site such as the skin and mucosa, and the difference is that the microbiome also includes the environment they inhabit or the collective genomes (12). The Common Fund Human Microbiome Project highlighted the interactions between microbiomes and human health issues such as T2D (13). Humans and microbes have a symbiotic relationship and a long history of shared ancestry (14). In the human body, the ratio of bacteria to human cells is close to 1:1 (15). Almost 65% of the human genome comes from microorganisms (16).

The human gut, or gastrointestinal tract, is the body’s largest digestive and immune organ. Each part of the gut has distinct characteristics. For example, the distal colon has the densest and the most diverse bacteria (17, 18). Gut microbiota has approximately 1,000 microbial species. The human gut microbiome comprises almost 10 million genes, which are more than 150 times the size of the human genome, and includes many metabolic genes (19). Human gut microbiota are mostly dominated by phylum of the phyla Firmicutes, Bacteroidetes, Actinobacteria, Proteobacteria, and Verrucomicrobia (18). Gut microbiota is highly involved in fighting against disease-causing microbes (e.g., promote IgA secretion) and energy metabolism (e.g., produce monosaccharides). The composition and proportion of gut microbiota are affected by genes [FXR (20)], lifestyle [diet (21)], drugs [antibiotics (22)], aging (23), etc. In addition, the gut microbiome is affected by the host feeding pattern (24), regulating circadian rhythms and metabolism in the host [reviewed in references (25, 26)]. In summary, gut microbiota plays an important role in human health and disease, such as T2D [reviewed in references (27, 28)].

**Fig 1:**
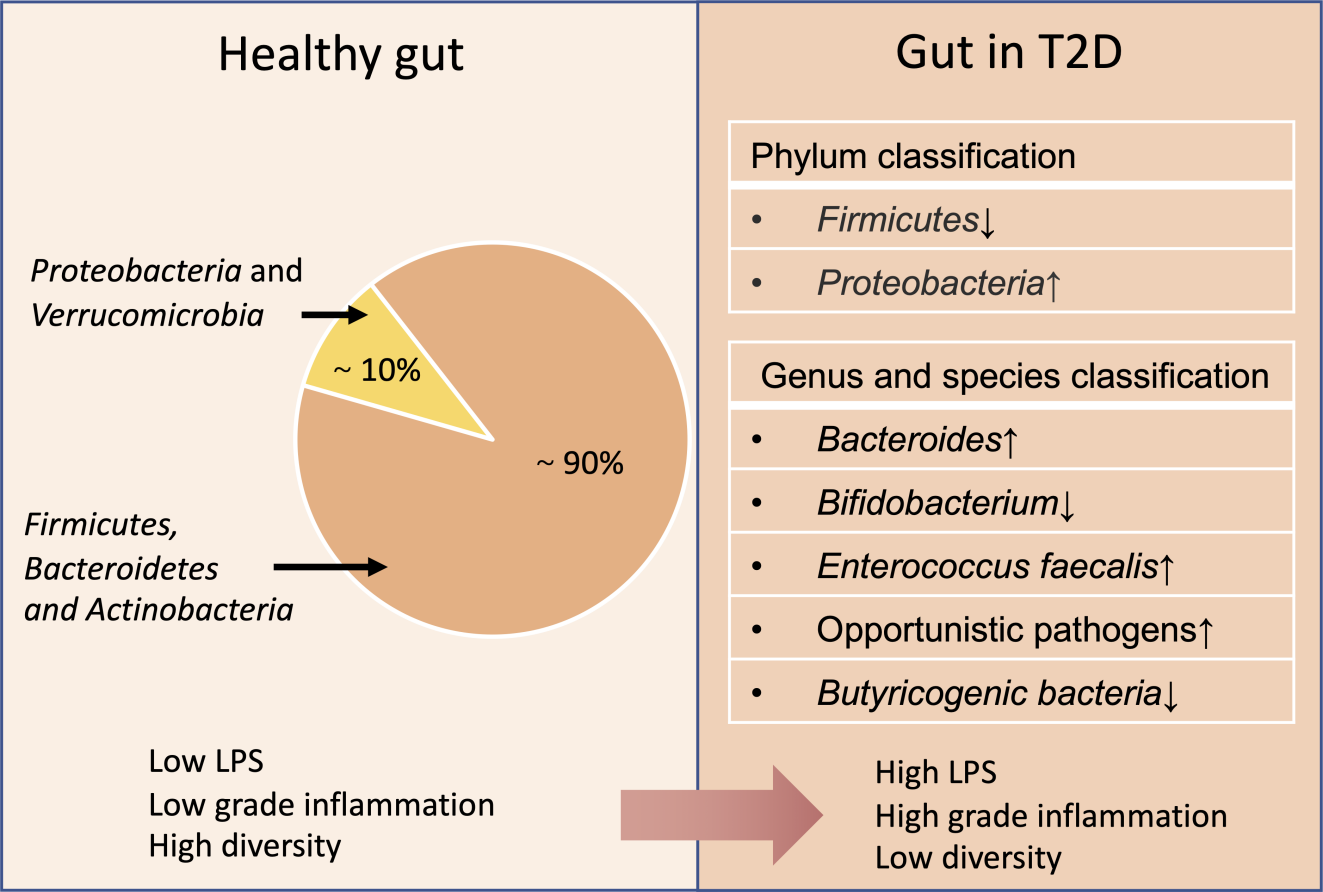
Comparison of intestinal flora between healthy and T2D subjects.

The gut microbiota is increasingly involved in the pathogenesis of diabetes. Meanwhile, both the composition and proportion of the gut microbiota are affected in patients with diabetes (Fig. 1). Patients with T2D had gut microbial dysbiosis and an increase in various opportunistic pathogens (29). Specifically, the abundance of some butyrate-producing bacteria (such as the phylum Firmicutes and genus *Bifidobacterium*) was reduced, while Gram-negative bacteria were relatively enriched such as the phyla Bacteroidetes and Proteobacteria (30, 31). However, alterations in certain gut microbiota by drugs or dietary fibers [such as decreased abundance of Firmicutes and Bacteroidetes (32)] contributed to the elevation of GLP-1 levels and the improvement of obesity-induced insulin resistance. Importantly, when discussing the influence of gut microbiota on the host, the individualization of host baseline microbiota and host variables should not be ignored (33, 34). In addition, comparative studies using germ-free mouse models demonstrated the importance of the gut microbiota in regulating diet-induced dysregulation of energy homeostasis and obesity (35, 36).

In this review, we summarized the interventions that might affect gut microbiota and then the secretion of GLP-1. Additionally, we reviewed the potential mechanisms in this process. Then, we highlighted the effect of gut microbiota on GLP-1 function and rhythm. Finally, the importance of gut peptides on gut microbiota was discussed. The interaction between gut flora and gut peptides provides a personalized approach to treat obesity and T2D.

## MECHANISMS BY WHICH GUT MICROBIOTA METABOLITES STIMULATE GLP-1 SECRETION

**Fig 2:**
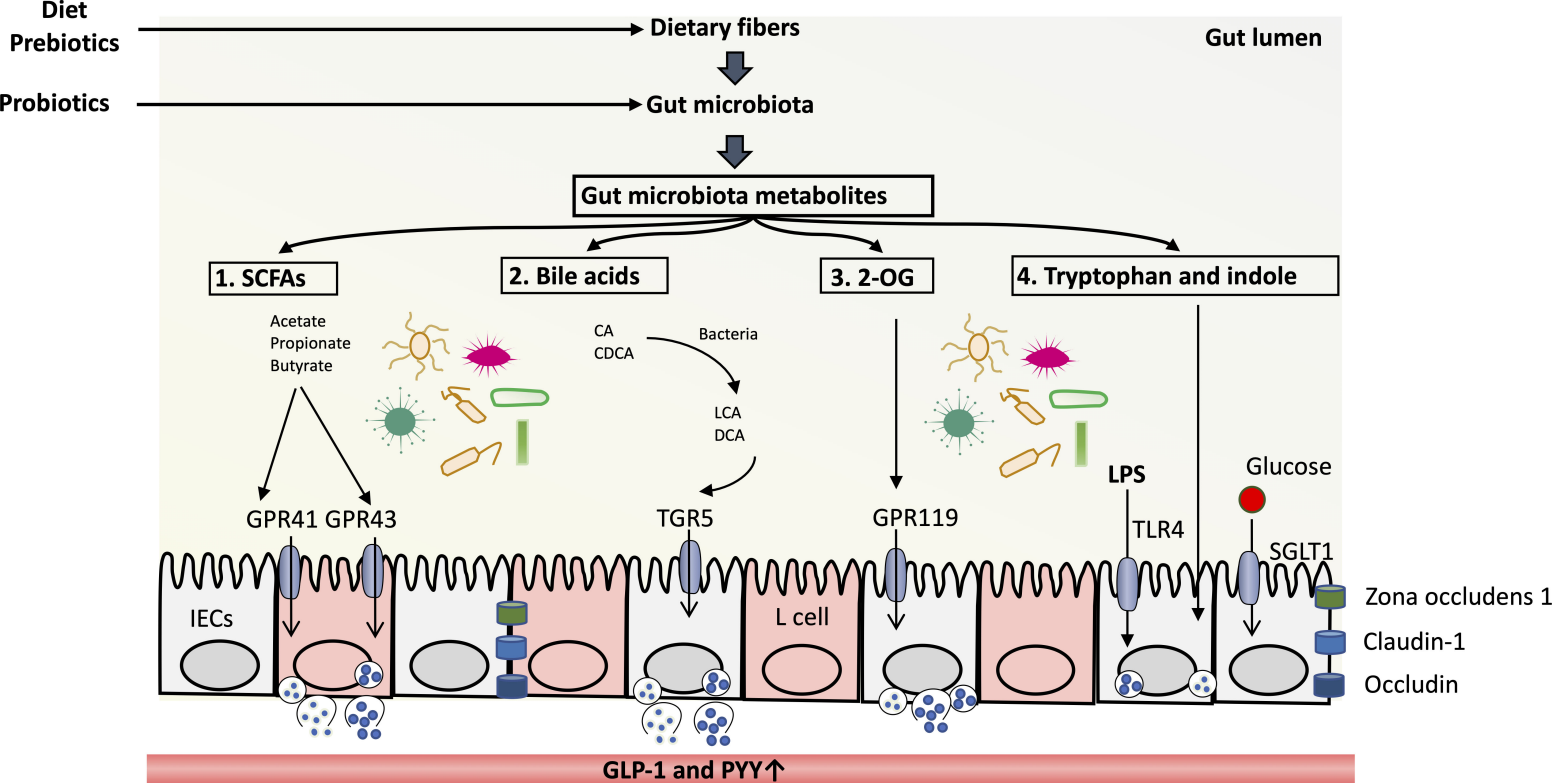
Gut microbiota metabolites promote GLP-1 production. The intake of prebiotics and certain diets produces dietary fibers, and the intake of probiotics may affect the function and composition of gut microbiota. (1) Dietary fibers are fermented to SCFAs by gut microbiota, which bind to GPR43 receptors on the surface of L cells and then promote the production of GLP-1. SCFAs also bind to GPR41 of L cells and promoted the PYY production. (2) Primary bile acids (CA and CDCA) are transported from the liver to the intestinal lumen, through a series of metabolism such as hydrolysis and dehydroxylation of gut microbiota, and finally are transformed into secondary bile acids (LCA and DCA). Secondary bile acids bind to TGR5 and promote the production of GLP-1. (3) Dietary fats are digested into 2-OG and fatty acids under the action of intestinal bacteria. 2-OG bind to GPR119 receptor of L cells to promote the production of GLP-1. (4) Tryptophan is the digestive product of dietary protein and then further broken down into indole. They promote the production of GLP-1. In addition, LPS on the surface of Gram-negative bacteria can bind to TLR4 receptor of L cells and then promote GLP-1 production. At the same time, intestinal epithelial cells can sense the concentration of glucose in the gut lumen and initiate the secretion of GLP-1 when the concentration reaches a certain level. 2-OG, 2-oleoyl glycerol; CA, cholic acid; CDCA, chenodeoxycholic acid; DCA, deoxycholic acid; GPR119, G-protein receptor 119; GPR41, G-protein receptor 41; GPR43, G-protein receptor 43; IECs, intestinal epithelial cells; LCA, lithocholic acid; LPS, lipopolysaccharides; SCFAs, short-chain fatty acids; SGLT1, sodium-glucose cotransporters 1; TGR5, Takeda G protein-coupled receptor 5; TLR4, Toll-like receptor 4.

Gut microbiota affects host GLP-1 production through metabolites (37). Several metabolites have been suggested to be involved in the influence of intestinal flora on GLP-1 secretion, as discussed below (Fig. 2).

Gut microbiota produces a variety of metabolites [including 5-HT (38), short-chain fatty acids (SCFAs) (39), secondary BAs (40). and lipopolysaccharide (LPS) (41)] that regulate enteroendocrine cells (EECs) and then the expression and secretion of hormones. Microbial metabolites can be divided into three categories. The first type, such as short-chain fatty acids and 2-oleoyl glycerol (2-OG, derived from dietary fats), is produced by intestinal microorganisms directly digesting or fermenting food components. The second category is metabolites produced by the host and modified by intestinal microorganisms, such as secondary BAs. Secondary BAs are dissociated and transformed from primary BAs by intestinal 7-α/β dehydroxylation bacteria and contribute to the establishment of intestinal homeostasis in hosts. The third type is the metabolites synthesized by intestinal microorganisms, such as LPS. Gut microbiota metabolites such as SCFAs (42–56) and secondary BAs (11, 57) can stimulate GLP-1 secretion. In addition, microbial metabolites such as 2-OG (58) and indole (59) directly activate GLP-1 secretion from L cells. Therefore, prebiotics and probiotics may ameliorate obesity and T2D through the gut microbiota-SCFA-inflammation/GLP-1 mechanism. Bariatric surgery may improve body weight, glucose metabolism, and inflammation by the gut microbiota-secondary BA-GLP-1 mechanism (11).

### SCFAs stimulate GLP-1 secretion

**Fig 3:**
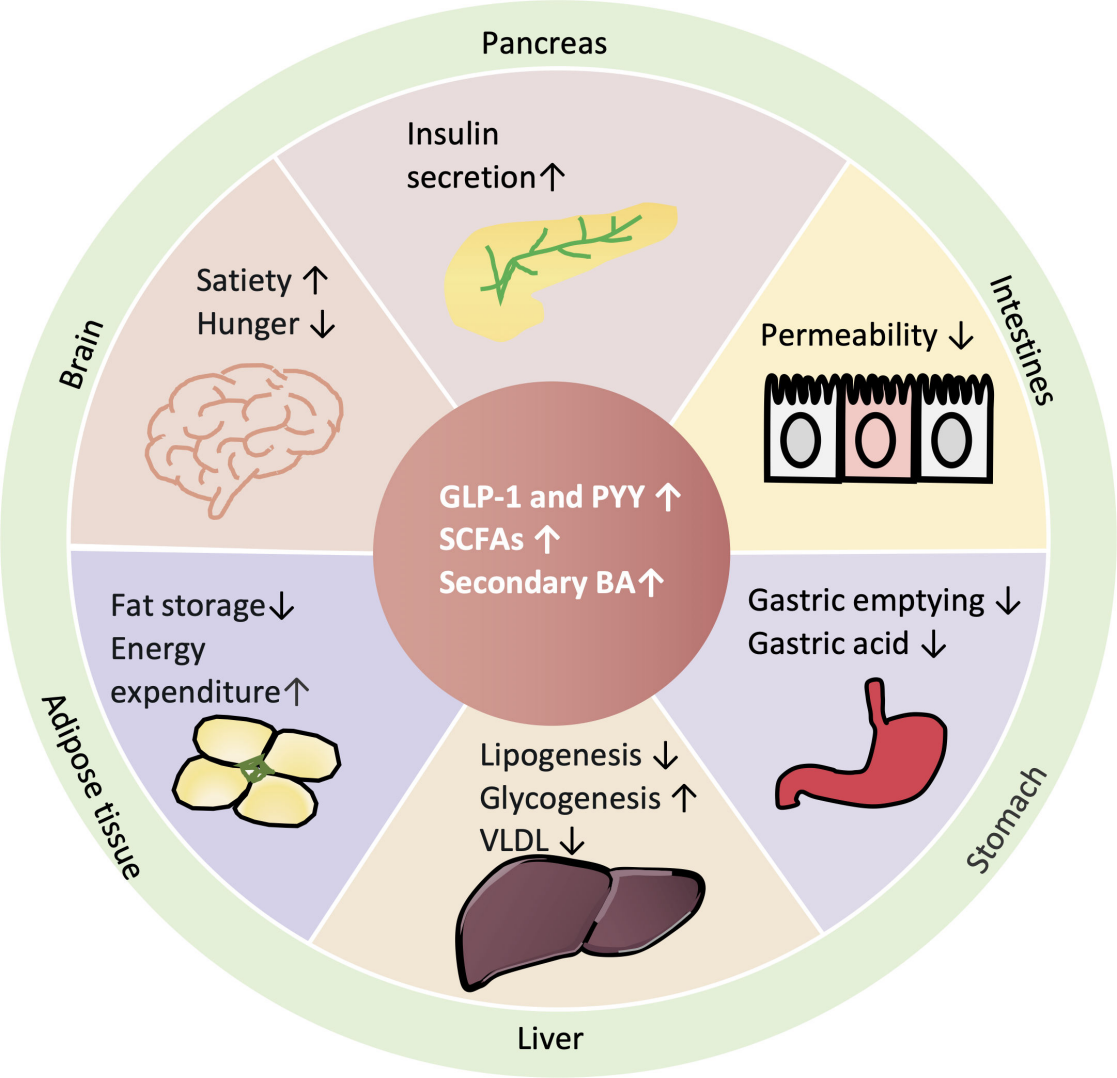
Physiological benefits of gut peptides and gut microbiota metabolites. GLP-1 has multiple physiological functions. It promotes insulin synthesis and secretion in pancreatic β cells and then improves glucose homeostasis, delays gastric emptying and reduces gastric acid secretion, reduces intestinal permeability and bacterial translocation, promotes lipolysis and energy expenditure, increases liver glycogen storage and decreases liver sugar output, and suppresses appetite in the hypothalamus of brain. SCFAs and secondary bile acids also help increasing insulin secretion in pancreas, energy expenditure in adipose tissue, and decreasing liver lipogenesis and VLDL (very low density lipoprotein) output.

SCFAs are involved in maintaining health and the development of disease and have attracted considerable attention. In fact, decreased SCFA production or production potential is associated with metabolic diseases, such as T2D (60, 61). The human genome encodes fewer than 20 enzymes to digest complex carbohydrates (62), so some carbohydrate polymers (dietary fibers) that are neither digested nor absorbed in the small intestine will be fermented to SCFAs by gut microbiota through carbohydrate-active enzymes in the gastrointestinal tract [reviewed in reference (63)]. Interestingly, the colon produces high levels of SCFAs and contains many L cells (64). More than 90% of SCFAs are absorbed by the gut or used by the microbiota (65). SCFA receptors are the G-protein-coupled free fatty acid receptors GPR43 (FFAR2) and GPR41 (FFAR3) (66). GPR43 and GPR41 are relatively conserved and highly expressed in enteroendocrine L cells in rats and humans and differ in their intracellular signals [reviewed in reference (67)]. In the human and rat colon and terminal ileum, the increase in SCFAs after adding fermenting dietary fiber may activate GPR43 and lead to increased GLP-1 secretion (51). Additionally, intravenous or rectal SCFA infusion was shown to increase GLP-1 secretion in humans (44). However, when SCFAs are given to GPR43 knockout mice, GLP-1 secretion cannot be stimulated (39). The mechanism involves GPR43 and GPR41 activation leading to increased intracellular calcium in L cells (39, 56, 68). Thus, these results suggest that gut microbiota can influence the production of SCFAs and the secretion of anorexic intestinal hormones, such as GLP-1, from rodent (39, 56) and human (44) enteroendocrine L cells via the receptor GPR43, but further studies are needed to elucidate the underlying mechanisms. However, GPR43 activation by increased SCFAs increased the number of the PYY-producing cells and PYY expression, which might be an effective therapeutic target for obesity but not T2D (69). Therefore, the increase in GLP-1 might occur through the receptor GPR41 (Fig. 3).

### Secondary bile acids stimulate GLP-1 secretion

Secondary BAs occur under the action of the gut microbiota, which means that alterations in the gut microbiota may change the composition of the BA pool. BAs as metabolites regulate signaling and glucose homeostasis. For example, secondary BAs have dual regulatory effects on GLP-1 secretion. On the one hand, secondary BAs activate Takeda G protein-coupled receptor 5 (TGR5) on intestinal L cells to stimulate GLP-1 secretion (70, 71). On the other hand, secondary BAs activate the farnesoid X receptor (FXR) to inhibit GLP-1 secretion (40, 72).

## INTERVENTIONS THAT AFFECT GUT MICROBIOTA TO PROMOTE GUT PEPTIDE SECRETION, SUCH AS GLP-1

Probiotics and prebiotics are beneficial for improving host health. They can modulate immune function, interact with the hosts’ gut microbiota, improve the gut barrier and permeability, and promote GLP-1 secretion.

### Prebiotics promoted GLP-1 secretion

Prebiotics can be selectively utilized by the host microbiota to improve host health (73). Recent findings suggested that prebiotic interventions lead to gut microbiota shifts to promote health (74). Prebiotics under the fermentation of gut microbiota could increase gut peptide production, such as GLP-1 and PYY (75).

In overweight/obese humans, supplementation with prebiotics such as oligofructose (76–79), fructan (75), resistant starch (42, 80), and arabinoxylan-oligosaccharide (81) has produced inconsistent results on GLP-1 and promoted SCFAs production (42, 78, 80–82) (Table 1). Of note, some oligofructose studies only found an increase in PYY, not GLP-1 (77, 78). Arabinoxylan-oligosaccharide caused a decrease in early postprandial GLP-1 accompanied by a decrease in alpha diversity and an increase in fecal *Bifidobacterium*, *Akkermansia*, and *Lactobacillus* (81). In patients with hyperinsulinemia, dietary fiber increased the production of acetate and butyrate to stimulate an increase in plasma GLP-1 and fasting and postprandial insulin levels, but the body weight stayed the same (43). An almond-based low carbohydrate diet consumption significantly increased the relative abundance of SCFA-producing bacteria *Roseburis* and *Ruminococcus* in human gastrointestinal microbiota (83, 84), as well as the GLP-1 concentration (84). Overall, human interventions with prebiotics have shown mixed results, so further work is needed.

**TABLE 1:** Clinical studies on the interaction between gut microbiota therapy and gut peptides^*a*^

Study design and references	Subject state or condition	BMI (kg/m^2^)	Intervention	Gut microbiota	Gut microbiota metabolites	Gut peptide	Outcomes
Crossover (76)	GERD (*n* = 9)	N/A	Oligofructose (20 g/d) for a week	N/A	N/A	GLP-1↑; PYY (-)	Breath H2↑; the rate of TLESRs↑
Double blind, R, parallel, PC (75)	Healthy (*n* = 10)	21.6 ± 0.99	Fructan (16 g/d) for 2 weeks	N/A	N/A	GLP-1↑; PYY↑	PBG↓; breath H2↑; hunger↓
Single blind, R, crossover (80)	T2D (*n* = 17)	31.0 ± 1.3	RS (40 g/d) for 12 weeks	N/A	Fasting serum propionate and butyrate↓	Fasting GLP-1↓; GLP-1↑	PBG↓; TAG↓; TNF-α↓; fasting NEFA↓; leptin or adiponectin (-)
R, crossover (42)	Healthy (*n* = 20)	23.6 ± 2.3	RS (17.0 g/d) and NSP (20.6 g/d) for 3 days	N/A	Fasting s-SCFA↑, (especially acetate↑)	Fasting GLP-1↑; PYY and GLP-2↑; OXM and ghrelin (-)	Breath H2↑; insulin sensitivity↑; blood glucose ↓; hunger, NEFA, and adiponectin (-)
Double blind, R, PC, P, trial (81)	Slow GI transit (*n* = 48)	24.7 ± 3.1	Axos (15 g/d) for 12 weeks	Alpha diversity↓; fecal *Bifidobacterium*↑, *Akkermansia*↑, *Lactobacillus*↑	Fecal and serum SCFA (-)	Early postprandial GLP-1↓; PYY (-)	Stool consistency↓; gut permeability/inflammation (-); glucose, insulin, FFA, TAG, glycerol; and appetite, hunger, satiety, and fullness ratings (-)
Double blind, R, placebo-controlled trial (77)	Overweight/obese (*n* = 39)	30.4 ± 3.4	FOS (21 g/d) for 12 weeks	N/A	N/A	GLP-1 (-), PYY↑; ghrelin↓;	Postprandial insulin↓; fat mass↓; energy intake↓; postprandial glucose (-); lipids (-);
Single blind, R, P, C study (78)	Healthy (*n* = 22)	29.7 ± 1.0	FOS (30 g/d) for 6 weeks	N/A	s-SCFA↑; acetate↑	GLP-1 (-); plasma PYY↑	Breath H2↑; appetite↓; hunger↓; fullness ↑; PBG, insulin (-); lipids, body fat (-); AST, ALT (-);
Double blind, R, crossover (79)	Healthy (*n* = 31)	24.8 ± 0.3	FOS (16 g/d) for 13 days	N/A	N/A	GLP-1↑; PYY↑	Energy intake↓
RCT (43)	FPI ≥40 pmol/L (*n* = 40)	25·7 ± 1.1	24 g fiber /d for a year	N/A	Acetate and butyrate↑	Plasma GLP-1↑	Body weight (-); fasting and postprandial insulin (-); NEFA (-)
Double blind, RCT (85)	NAFLD children (*n* = 44)	27.3 (24.7–28.6)	VSL#3 for 4 months	N/A	N/A	GLP-1 and activated GLP-1↑	BMI↓
Double blind, R trial (86)	Glucose tolerant (*n* = 21)	23.6 ± 1.7	*L. reuteri* (2 × 10^10^ cells b.i.d.) for 4 weeks	a-Diversity, overall composition, and total lactobacilli (-);	N/A	GLP-1↑; GLP-2↑	Insulin↑; C-peptide↑; PBG (-); IL-8, and MIP-1b (-); TNF-α↑; oxidative stress (-)
Double blind, R, PC crossover study (57)	MetS (*n* = 12)	35.9 (32.3–37.9)	Duodenal infusion *A. soehngenii* L2-7 treatment	Microbiota richness and diversity (-); fecal SCFA (-)	Plasma secondary BA (TDCA, TLCA, GDCA); butyrate↑	GLP-1↑	GPR43, TGR5, FXR5, and REG1B↑
Single blind, R, crossover (44)	Hyperinsulinemic female (*n* = 6)	31·0 (SEM 1.0)	Rectal or intravenous acetate infusions	N/A	Plasma acetate↑; Cecal SCFA (-)	GLP-1↑; PYY↑ ghrelin (-)	Plasma glucose or insulin (-); TNF-α and NEFA (-)
Clinical trial (87)	T2D (*n* = 14)	30.0 ± 3.3	Stopping metformin	Firmicutes↑; Bacteroidetes↓	Cholic acid and conjugates↑	GLP-1↓	Plasma glucose levels↑
RCT (88)	T2D (*n* = 19)	33.3 ± 4.1	Metformin 1,000 mg twice daily	N/A	N/A	GLP-1R; PYY↑	PBG↓; fasting glusocse levels↓
R, open-labeled, two-arm trial (89)	Treatment-naive T2D (*n* = 92)	26.83 ± 1.81	Acarbose or vildagliptin for 6 months	Bacteroidetes species↓	N/A	Fasting GLP-1↑	HbA1c↓; visceral fat areas
Clinical trial (45)	Diagnosed-naive diabetes (*n* = 50)	25.82 ± 2.88	Metformin 1,500 mg/d for 12 weeks	*Phascolarctobacterium*, *Intestinimonas,* and *Clostridium* III↑	Acetic acid and propanoic acid in feces↑	Total GLP-1↑	Food intake↓; glucose control↑
Observational study (90)	Morbidly obese (*n* = 3)	40.6 ± 5.4	RYGB surgery	Firmicutes↓; Gammaproteobacteria↑; Archaea↓	N/A	N/A	N/A

^*a*^ ALT, alaninetransaminase; AST, aspartate transaminase; *A. soehngenii* L2-7, *Anaerobutyricum soehngenii* L2-7; Axos, arabinoxylan-oligosaccharide; BMI, body mass index; C, controlled; FOS, oligo-fructose; FPI, fasting plasma insulin; GDCA, glycodeoxycholic acid; GERD, gastro-oesophageal reflux disease; *L. reuteri*, *Lactobacillus reuteri*; MetS, metabolic syndrome; NAFLD, nonalcoholic fatty liver disease; NEFA, non-esterified fatty acids; NSP, non-starch polysaccharides; OXM, oxyntomodulin; P, parallel; PBG, postprandial blood glucose; PC, placebo controlled; R, randomized; REG1B, regenerating islet-protein 1B; RS, resistant starch; RYGB, Roux-en-Y gastric bypass; SEM, standard error of mean; s-SCFA, serum SCFA; TDCA, taurodeoxycholic acid; TLCA, taurolithocholic acid; TLESRs, transient lower esophageal sphincter relaxations. Values expressed as mean ± SD or medians and IQRs. ↑, increase; ↓, decrease; (-), no change; NA, not available.

The effects of prebiotics on GLP-1 secretion are also inconsistent. In mice, ingestion of prebiotics increased butyrate-producing bacteria (48), enhanced GLP-1 release (91), and improved diabetes symptoms (92). For example, dendrobium polysaccharides upregulated the abundance of *Akkermansia* and *Parabacteroides*, thereby increasing gut microbiota metabolites such as SCFAs, tryptophan, and indole to stimulate GLP-1 secretion [reviewed in reference (93)]. In diabetic mice, ingestion of resveratrol enhanced GLP-1 release and modified cecal bacterial composition (94). In db/db mice, tetrahydrocurcumin supplementation decreased the ratio of Firmicutes to Bacteroidetes and increased the volume of GLP-1 in the pancreas (95). Oligofructose (49), fructo-oligosaccharide (51), fructans (96), and inulin (97) caused an increase in GLP-1, PYY, and SCFAs, but the change in gut microbiota was not applicable. In T2D mice, the supplementation of modified dietary fibers increased the relative abundance of *Akkermansia muciniphila*, Verrucomicrobia*,* and Bacteroidetes, decreased the relative abundance of Firmicutes, Proteobacteria, and Actinobacteria, and increased the production of SCFAs. It also increased the levels of GLP-1 and PYY and then improved the metabolism of blood glucose and lipids (46). Additionally, flavonoids from *Lycium barbarum* regulate the gut microbiota and reduce pro-inflammatory cytokines to ameliorate the symptoms of T2D mice, accompanied by the elevation of GLP-1 (98). Polysaccharides from adlay seeds (PAS) increased Simpson’s diversity index and GLP-1 concentrations, indicating that PAS altered the diversity and composition of the microbiota and had hypoglycemic effects in T2D mice (99) (Table 2). In conclusion, one of the mechanisms by which prebiotics regulates host health is to promote GLP-1 secretion by regulating changes in gut microbiota.

**TABLE 2:** Animal studies on the interaction between gut microbiota modification therapy and gut peptides^*a*^

Animal model and references	Invention	Gut microbiota	Gut microbiota metabolites	Gut peptide	Metabolism	Putative outcomes
Male C57Bl/6J diabetic mice (94)	Resveratrol diet (60 mg RSV/Kg/day) for 5 weeks	Modified cecal bacterial composition	N/A	Active GLP-1 in the colon↑; portal vein GLP-1↑	Insulin; proglucagon mRNA↑	N/A
db/db Mice (95)	THC for 8 weeks	Proteobacteria, Actinobacteria, and F/B ratio↓	N/A	GLP-1 in the pancreas↑;	FBG↓; insulin↑	Islet injury↓
T2D male Kunming mice (47)	Kombucha for 4 weeks	SCFAs-producing bacteria↑; Gram-negative bacteria and pathogenic bacteria↓; Firmicutes↑; Proteobacteria↓	SCFAs↑, especially butyric acid and acetic acid	GLP-1↑; PYY↑; GPR41 and GPR43↑;	FBG↓; food intake↓; BW↑; HOMA-IR↓; glycogen synthesis↑; AST, ALT, and the liver coefficient↓	LPS↓; islet cells↑; the pancreatic index↓; IL-1β, IL-6, and TFN-α↓; colonic injury recovered; tight junction proteins and mucin↑
T2D male C57BL/6 J mice (46)	Modified DFs for 4 weeks	*Akkermansia muciniphila*↑; Verrucomicrobia and Bacteroidetes↑; Firmicutes, Proteobacteria, and Actinobacteria↓; F/B ratio↓	SCFAs↑, especially acetic acid, propionic acid, and butyric acid	GLP-1↑; PYY↑	FBG↓; insulin and leptin↑; liver to body ratio↓; TC, TG, and LDL-C↓; HDL-C↑; pancreatic islets↑; liver injury↓	Glut2 and insulin receptor in the liver↓; G6Pase↑
Diabetic mice (98)	LBFs	N/A	N/A	GLP-1↑; TLR-4↓	FBG↓; HOMA-IR, HOMA-IS, and HbA1c↓; OGTT↓; TC and TG↓	LPS, TLR-4, TNF-4, IL-6, IL-10↓
T2D ICR male mice (99)	PAS	Altered the diversity and composition of the microbiota	N/A	GLP-1↑	BG↓; HbA1c↓; TC and TG↓; AC1-42↓; STZ-lesioned pancreatic cells↓	N/A
Goto-Kakizaki rat (48)	An RS diet for 10 weeks	Butyrate-producing bacteria in cecal contents↑	SCFAs↑ in cecal contents	Total GLP-1↑	FBG↓; fasting insulin↓; pancreatic β cell mass↑; insulin sensitivity↑; pancreatic insulin content↑; fat weight↓	N/A
Male Wistar rats (49)	Oligofructose (10 g/100 g diet) for 4 weeks	N/A	Butyrate↑ in the cecum and proximal colon	Portal serum GLP-1↑	Food intake, energy intake, and body weight gain↓	Enteroendocrine L-cells↑; neurogenin 3 and NeuroD↑; total cecum weight↑
C57J/B6 male mice and Lep receptor-deficiency ob/ob mice (50)	VSL#3 for 8 weeks	Butyrate-producing bacteria↑	Butyrate in the fecal and serum samples↑	GLP-1↑; FFAR3↑	FBG↓; glucose tolerance and insulin tolerance↑	Genes involved in GLP-1 synthesis (Gcg and Pcsk1) and secretion (Slc5a1) ↑
HFD-fed male C57BL/6 J mice (91)	FOS for 4 weeks	N/A	N/A	GLP-1↑	Glucose tolerance↑, FBG↓; Insulin↑, and body weight gain↓	Hepatic phosphorylation of IKK-beta and NF-B↓
Male Wistar rats (51)	Fructo-oligosaccharide (16 g/day) for 28 days	N/A	SCFA in colon and terminal ileum↑, especially acetate and butyrate	GLP-1↑	Densities of FFA2- and GLP-1-IR cells↑;	The weights of the cecal tissues and contents↑
Male Wistar rats (96)	Fructans (100 g) for 3 weeks	N/A	N/A	Portal vein serum GLP-1↑; ghrelin↓	Epididymal fat mass↓	N/A
WT (97)	Inulin (for 2 or 14 weeks)	N/A	SCFAs↑	PYY↑	BW gain↓; IR↓; food intake↓; glucose tolerance↑	N/A
DIO rats (100)	*L. paracasei* intervention for 3 or 12 weeks	Composition of the cecum microbiome (-)	N/A	GLP-1↑	Serum LDL-C↓; TG↓; insulin secretion↑; IR index (-); weight gain (-); TRL-C↓; BG↓; fasting cholesterol↓; MAT↓; EAT↓;	GLP-1 intervention; serum molecular signature changed; microbiome mediated
Male db/db diabetic mice (52)	10 *Lactobacillus* strains and 4 *Saccharomycetes* strains composite probiotics (6 wks)	Bacteroidetes/*Bifidobacterium*/*Lactobacillus*/*Clostridium leptum*/*Roseburia,* and *Prevotella*↑; Firmicutes/ Actinobacteria/*Enterococcus faecium*/Gram-negative bacteria/*Escherichia coli* and *Bacteroides thetaiotaomicron*↓	Propionate and butyrate↑; acetate↓	GLP-1↑; PYY↑ GPR43↑; GPR41↑	PBG↓; C-peptide↑; TG, TC, and LDL-C↓; insulin↑	Improved pancreas function, immune state, and the intestinal barrier function
Male C57BL/6J diabetic mice (53)	*L. casei* CCFM419	Bacteroidetes/*Bifidobacterium*/*Lactobacillus*/ SCFA-producing bacteria↑; Firmicutes↓	Acetic acid, butyric acid, and total SCFAs in the feces↑	GLP-1↑	PBG↓; HbA1C↓; leptin↓; LDL-C↓; HDL-C↑;	TNF-α↓; IL-6↓
Leptin receptor-deficiency db/db mice (54)	Oral gavage CB0313.1 daily (5 weeks)	N/A	N/A	N/A	Insulin sensitivity↑; improved glucose tolerance	Inflammatory tone in adipose tissue↓
C57BL/6J diabetic mice (54)	Oral gavage CB0313.1 daily (13 weeks)	Butyrate-producing bacteria↑	SCFA↑; SCFA receptor↑	GLP-1↑	Insulin sensitivity↑; glucose tolerance↑; HOMA-β↑	Inflammatory tone in adipose tissue↓; TNF-α↓; MCP-1↓
Female Wistar diabetic rats (101)	Oral *L. fermentum* MCC2759/2760 (4 weeks)	Pathogenic bacteria such as *Escherichia coli*, *Staphylococcus aureus*, and *Campylobacter* spp. ↓	N/A	GLP-1↑; TLR4 receptor↓	Glucose tolerance↑; plasma insulin↓; BG↓; adiponectin↑; GLUT4↑	Tight junction protein ZO-1↑; endocannabinoid receptor CB2↑;
Male albino Wistar T2D rats (55)	*L. rhamnosus* NCDC 17 (9.5 to 10 log cfu/mL) (6 weeks)	Bacteria abundance and numbers↑; *Eubacterium rectale*-*Clostridium coccoides*, *Bacteroides*, *Lactobacilli,* and *Bifidobacteria*↑ (cecal contents)	Acetate↑	GLP-1↑	Glucose tolerance↑; FBG↓; HDL-C↑; TG↓; VLDL-C↑; adiponectin↑	Activity of catalase and GPx↑; activity of SOD↑；TNF-v↓ and IL-6↓;
Male albino Wistar T2D rats (55)	*L. rhamnosus* LGG (8 to 8.5 log cfu/mL) (6 weeks)	Bacteria abundance and numbers↑; *Bacteroides*↓ (cecal contents)	Propionate↑	GLP-1↑	Glucose tolerance↓; FBG↓; HDL-C↑;	Activity of catalase and GPx↑；activity of SOD↑
ICR mice (102)	Oral EPSs (800 mg/kg)	N/A	N/A	GLP-1↑	BG↓; glucose consumption of the FL83B cells↑	Activation of Akt↑
HCD-fed C57BL6 mice (103)	Oral *L. fermentum* MCC2760 (10.95 log CFU/mL) (8 weeks)	*Lactobacillus* spp. count↑; pathogen count (like *Staphylococcus* and *Campylobacter*)↓	N/A	GLP-1↑	BG↓; body weight↓; serum CHOL, TG, LDL-C, AST, and ALT↓	Bacterial translocation count↓; LPS, TNF-α, IL-6, IL-12↓. and IL-10; GSH-Px, GSH-Tr, CAT, and SOD↑; CB1↓, CB2↑; ZO-1↑
Piglets (104)	*Lactobacillus plantarum* (50 mg) (3.5 × 10^10^ CFU/g) daily (2 weeks)	Alpha diversity (-); Tenericutes phylum/ *Bacteroides*/*Parabacteroide*/*Clostridium_sensu_stricto*_1/*Ruminococcus*_1/*Desulfovibrio*↓; *Lactobacillus*/*Megasphaera*/*Collinsella*↑	G-LCA↓; T-LCA↓; CA↑; TBA (-) (in ileum tissue)	Postprandial plasma GLP-1↓	PBG↑; genes associated with BA metabolism (-)	Genes related to inflammation and glucose transport (-); GLUT2 and SGLT1 (-)
Pig (105)	Cecal propionate infusions (0, 5, 20, and 100 mmol/L)	N/A	Exogenous propionate↑	GLP-1↑; PYY↑; FFAR2/FFAR3 expression↑	Acute feed intake↓;	AgRP expression↓
FFAR2 (−/−) mice (56)	Colonic propionate infusions (180 mmol/L)	N/A	Exogenous propionate↑	Portal vein plasma GLP-1 and PYY (-)	N/A	N/A
Wistar rats and C57BL6 mice (56)	Colonic propionate infusions (180 mmol/L)	N/A	Exogenous propionate↑	Portal vein plasma GLP-1 and PYY↑	N/A	N/A
FFAR2^−/−^ and FFAR3^−/−^ mice (39)	Receptor knockout (3 to 4 months)	N/A	SCFAs↑	GLP-1↓	N/A	N/A
Male HFD-fed C57BL/6 J mice (106)	FMT	*Akkermansia*, *Bacteroides*, and *Butyricimonas* (-)	N/A	GLP-1↑; TLR1 and TLR4↑	BG↑; body weight (-); TC (-);	IL-18↓; TLR2, TLR5, and TLR6 (-);
Male HFD-fed C57BL/6 J mice (107)	Metformin (250 mg/kg) for 16 weeks	Genera *Akkermansia*, *Bacteroides*, *Butyricimonas*, and *Parabacteroides*↑	N/A	N/A	BG↓; BW↓; TC and LDL↓	IL-1β and IL-6 in epididymal fat↓
DIO male C57Bl/6 J mice (11)	SG	*Clostridiales* (-)	LCA in portal veins↑	GLP-1↑	Expression levels of mSult2A1 and Vdr in liver↑; CA7S↑	LCA-VDR-SULT2A1-CA7S pathway
Male C57BL/6 J mice, SD rats, and Zucker diabetic fatty rats (108)	Canagliflozin (0.3–30 mg/kg) and sitagliptin (10 mg/kg)	N/A	N/A	Plasma active GLP-1↑	Insulin↑; BG↓	Transient intestinal SGLT1↓
Male C57BL/6 renal failure mice (109)	Canagliflozin (10 mg/kg for 2 weeks)	*Bifidobacterium*↓	Colonic SCFAs↑	N/A	p-Cresyl sulfate and indoxyl sulfate↓; BG (-)	Intestinal SGLT1↓

^*a*^ AgRP, agouti-related protein; ALT, alanine transaminase; AST, aspartate aminotransferase; BW, body weight; CA, cholic acid; CAT, catalase; CB0313.1, *Clostridium butyricum* CGMCC0313.1; CFU, colony-forming units; CHOL, cholesterol; DFs, dietary fibers; DIO, diet-induced obese; DOP, dendrobium polysaccharide; EAT, epididymal adipose tissue; EPSs, exopolysaccharides; FBG, fasting blood glucose levels; F/B ratio, the ratio of Firmicutes to Bacteroidetes; FMT, fecal microbiota transplantation; G6pase, glucose-6-phosphatase catalytic subunit 1; GB-IL, bile diversion to the ileum; G-LCA, glycolithocholic acid; GSH-Px, glutathione peroxidase; GSH-Tr, glutathione transferase; HCD, high-cholesterol diet; HDL-C, high density lipoprotein cholesterol; HOMA-IR, homeostatic model assessment of insulin resistance; HOMA-IS, homeostasis model assessment of insulin sensitivity; IL, interleukin; IR, insulin resistance; *L. fermentum*, *Lactobacillus fermentum*; *L. Paracasei*, *Lactobacillus paracasei*; LBFs, flavonoids from *Lycium barbarum*; LDL-C, low-density lipoprotein cholesterol; LPS, lipopolysaccharide; MAT, mesenteric adipose tissue; MCP-1, monocyte chemotactic protein 1; NSS, not statistically significant; PAS, polysaccharides from adlay seeds; PBG, postprandial blood glucose; RSV, resveratrol; SG, sleeve gastrectomy; SOD, superoxide dismutase; T1D, type 1 diabetes; TBA, total bile acids; TC, total cholesterol; TG, triglycerides; THC, tetrahydrocurcumin; T-LCA, total lithocholic acid; TLR, Toll-like receptor; TNF, tumor necrosis factor; TRL-C, triglyceride-rich lipoprotein cholesterol; VSL#3, a commercial product containing a total of eight probiotic strains including *Streptococcus thermophilus*, *bifidobacterium* (*B. breve*, *B. infantis*, *B. longum)*, *Lactobacillus acidophilus*, *L. plantarum*, *L. paracasei*, and *L. delbrueckii* subsp. *bulgaricus*; ZO, zonula occludens-1.↑, increase; ↓, decrease; (-), no change; NA, not available.

### Probiotics promoted GLP-1 secretion

Probiotics are live microorganisms that bring many benefits to the host (110). Probiotic interventions have strain-specific anti-inflammatory effects on healthy adults (111). While the effects of probiotics on the host are not necessarily related to their interactions with the protoflora, their use is often associated with claims about beneficial regulation of probiotics and the normalization of disturbed flora, either as a favorable outcome of the probiotics themselves or as a mechanism by which the probiotics protect the host against disease (73). However, the effect of probiotic intake on intestinal mucosa is not necessarily fixed and is related to the host and its microbiome characteristics (112). The beneficial effects of probiotics on diabetes have been studied, and the mechanism may be related to enhancing immunity, increasing the production of anti-inflammatory cytokines, reducing intestinal permeability, and reducing oxidative stress.

In subjects with metabolic syndrome, a single duodenal *Anaerobutyricum soehngenii* bacteria infusion increased the levels of plasma secondary BAs and postprandial GLP-1 and thereby improved glucose metabolism (57). Moreover, no adverse events were observed when live *Anaerobutyricum soehng*eni was orally administered (113). Intake of *Lactobacillus reuteri* improved GLP-1 and insulin secretion in people with glucose tolerance (86). Supplementation with VSL#3 (a commercial product containing a total of eight probiotic strains including *Streptococcus thermophilus*, *bifidobacterium* [*B. breve*, *B. infantis*, *B. longum*], *Lactobacillus acidophilus*, *L. plantarum*, *L. paracasei*, and *L. delbrueckii* subsp. *bulgaricus*) for 4 months increased GLP-1 and decreased BMI in nonalcoholic fatty liver disease children (85).

In T2D mice, Kombucha (polyphenols and organic acid active substances) administration improved the inflammation state and intestinal tight conjunction, such as decreasing the levels of LPS and pancreatic index and increasing the protein zona occludens 1, claudin-1, occludin, and mucin (47). At the same time, the abundance of SCFA-producing bacteria was increased, thereby increasing SCFAs and elevating the concentrations of GLP-1 and PYY (47). Exopolysaccharides from *Bacillus amyloliquefaciens* could increase GLP-1 levels by interacting with intestinal tissues (102). Supplementation with VSL#3 for 8 weeks increased the abundance of butyrate-producing bacteria, butyrate, and GLP-1 and improved fasting blood glucose, glucose, and insulin tolerance in both C57J/B6 male mice and Lep^ob/ob^ mice (50). A recombinant microbe *Lactobacillus paracasei* NFBC 338 was successfully transformed to express a long-acting analog of GLP-1. The short-term or long-term administration of *L*. *paracasei* NFBC 338 did not change the composition of the cecum microbiome but improved glucose or lipid metabolism in diet-induced obese (DIO) rats (100). Supplementation with composite probiotics stimulated the secretion of GLP-1 and PYY by changing the composition of the gut microbiota and the production of SCFAs. At the same time, the metabolism of blood glucose and lipids, immune state, and pancreas function were improved in db/db diabetic mice (52). In T2D mice, oral administration of *Lactobacillus casei* increased the abundance of Bacteroidetes, *Bifidobacterium*, and *Lactob*acillus, and butyrate production increased, which stimulated GLP-1 secretion (53). Daily administration of *Clostridium butyricum* CGMCC0313.1 for 13 weeks decreased Firmicutes/Bacteroidetes ratios and increased SCFA-producing bacteria and SCFA receptors FFAR2 and FFAR3 in T2D mice. Moreover, serum and ileal GLP-1 levels increased, but this improvement has not been observed in leptin receptor-deficient db/db mice (54). This may be related to the time of administration and the different mouse models. Supplementation with *L. fermentum* MCC2759/*L. fermentum* MCC2760 orally or intragastrically decreased the count of pathogenic bacteria and increased the production of GLP-1 and *Lactobacillus* spp. count (101, 103). Two different concentrations of L. rhamnosus LGG increased the bacterial abundance, number, and GLP-1 levels. However, the increased types of SCFAs were acetate or propionate (55). In piglets, the supplementation with L. *plantarum* reduced the abundance of *Bacteroides* and *Parabacteroides*, and also decreased the levels of lithocholic acid (LCA), eventually increasing BG (104). In conclusion, probiotics regulate host health by regulating changes in gut microbiota.

## EFFECT OF GUT MICROBIOTA ON GLP-1 FUNCTION

Incretin-based drugs are effective in treating individuals with diabetes. Many studies have demonstrated that the incretin effect is impaired in obesity, IGT (impaired glucose tolerance), and T2D patients. However, sometimes it is necessary for patients to stop their treatment with GLP-1 RA due to a lack of efficacy. This phenomenon is a state of GLP-1 resistance (114). This state could be caused by gut microbiota dysbiosis (115).

In a human study, different gut microbiota compositions have different responses to GLP-1 RA (116). T2D patients on a treatment with GLP-1 RA (liraglutide or dulaglutide) for 12 weeks were divided into GLP-1 RA responders (*n* = 34) and non-responders (*n* = 18). The former had both decreased levels of HbA1c and BMI, and the latter had no change in these two variables. The beta diversity of gut microbiota was significantly differed between these two groups, as well as some bacteria, such as *Bacteroides dorei* and *Roseburia inulinivorans*. So, the signature of gut microbiota may predict the GLP-1 RA efficacy. In 2017, how gut microbiota dysbiosis induces GLP-1 resistance was well exhibited in mice (117). In this study, two T2D mouse models were created: a diabetic obese model and a diabetic lean model. At 15 min after OGTT (oral glucose tolerance test) experiments, glycemia was almost similar between these two diabetic groups and normal control mice. However, in the diabetic lean model, the plasma GLP-1 concentration was higher, but the plasma insulin concentration was lower than that in the diabetic obese model and normal model. This suggested that GLP-1 resistance existed in the diabetic lean model. Then, the ileum microbiota was transplanted from the two diabetic groups and the normal control group to germ-free mice. The results demonstrated that the incretin effect was impaired, while GLP-1R expression was slightly higher in germ-free mice after fecal transplantation from diabetic lean mice compared to other groups. This result suggested that the function of GLP-1 was dependent on the normal gut microbiota. Gut microbiota dysbiosis impaired GLP-1 responsiveness (117). Therefore, the gut microbiota is closely responsible for GLP-1 function (89).

## EFFECT OF GUT MICROBIOTA ON GLP-1 RHYTHM

Circadian rhythms refer to physiological changes in an organism’s activities that occur almost every 24 hours, also known as the biological clock (118). This biological clock exists not only in the brain but also in peripheral organs, such as the pancreas and gastrointestinal tract. It has been proven that pancreatic islets have circadian genes such as CLOCK and BMAL1 in *Homo sapiens* and rodents (119, 120), and disruption of circadian genes leads to diabetes (121). Moreover, the secretion of insulin abides by a circadian clock pattern (122). In humans, it was revealed that GLP-1 secretion has temporal differences because early GLP-1 release was more prominent in the morning than in the afternoon (123). A significant circadian rhythm in GLP-1 secretion by intestinal L cells (124) or GLP-1 responsiveness (125) was found in animal experiments. In mice, the peak time of GLP-1 release was 8 p.m. (ZT14). The bottom time of GLP-1 secretion was 8 a.m. (ZT2). Taken together, there is clear evidence that GLP-1 secretion has a circadian rhythm. The composition and function of the gut microbiota also exhibit some oscillations that follow the host dietary pattern. In return, gut microbiota regulate host circadian rhythms and metabolism [reviewed in reference (26)].

**Fig 4:**
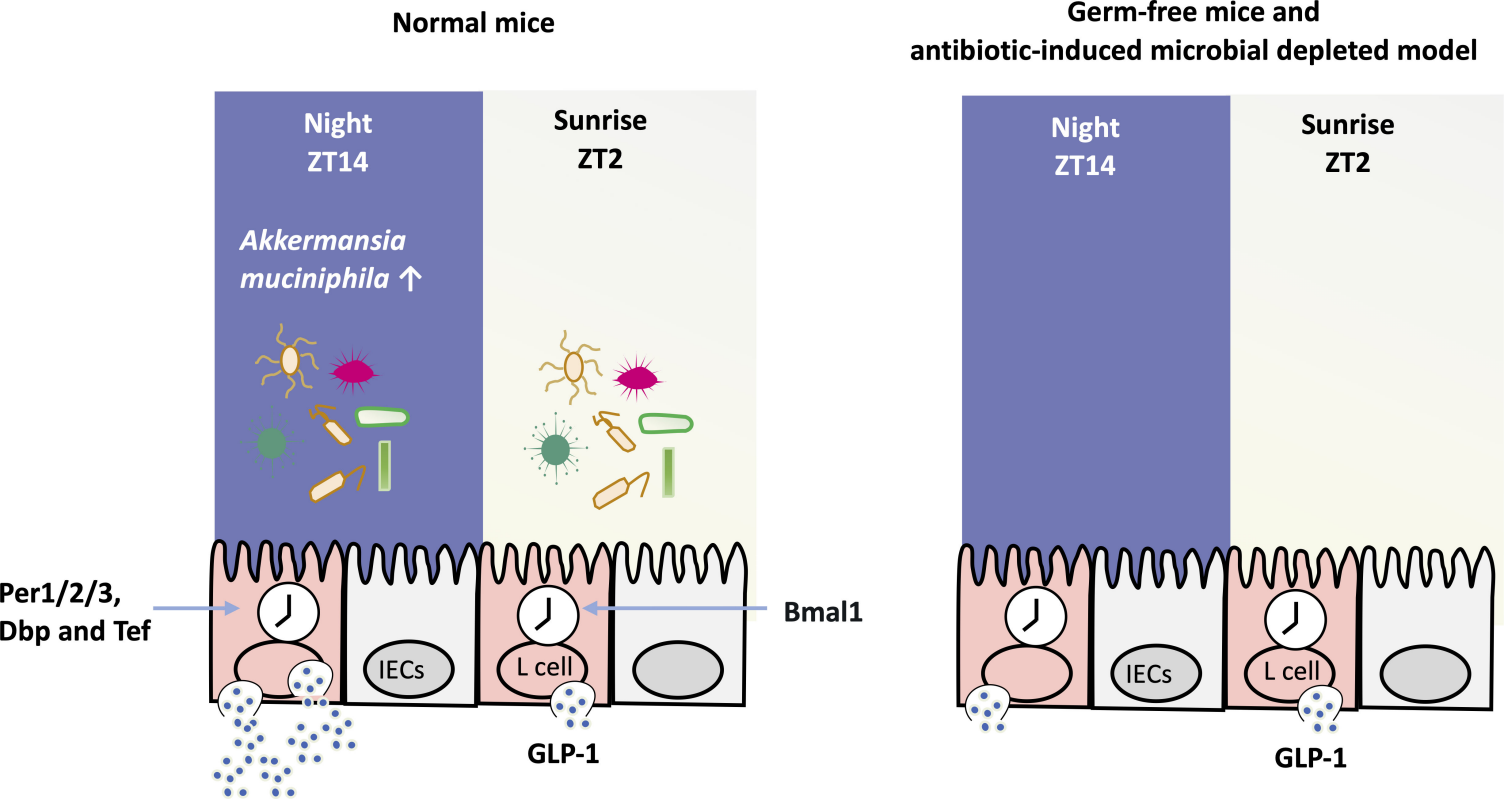
The circadian rhythm of GLP-1 release mediated by L cells and regulated by gut microbiota. GLP-1 secretion in normal mice showed a circadian rhythm, with the peak of 8 p.m. (ZT14) and the bottom line of 8 a.m. (ZT2). The abundance of *Akkermansia muciniphila* which was closely related to the secretion of GLP-1 was higher at ZT14 than at ZT2. While in germ-free mice and antibiotic-induced microbial depleted model, the GLP-1 rhythm was not exhibited. And the biological rhythms of L cells regulated GLP-1 release. The clock gene Bmal1 was significantly increased at ZT2. While Per1/2/3, Dbp, and Tef increased at ZT14.

The regulation of GLP-1 secretion rhythm by gut microbiota is essential. First, gut microbiota disorders can affect the rhythmic secretion of GLP-1. In germ-free mice without gut microbiota, there was no circadian rhythm of insulin secretion. However, after fecal transplantation from normal diet-fed mice, the insulin rhythm reappeared (126). Therefore, the homeostasis of the gut microbiota environment was significant for the rhythmic secretion of GLP-1 (126). Second, the same team demonstrated that the biological rhythms of L cells regulated GLP-1 release. The core biological clock gene Bmal1 in intestinal L cells regulates the rhythm of GLP-1 secretion (127), as do Per1/2/3, Dbp, and Tef (126). Knockdown of Bmal1 in L cells impaired GLP-1 circadian secretion (128). In summary, the circadian rhythm of GLP-1 release is mediated by L cells and regulated by the gut microbiota (Fig. 4).

## EFFECT OF GLP-1 ON GUT MICROBIOTA

### GLP-1 analogs and DPP-4 inhibitor changed the composition and abundance of gut microbiota

The GLP-1 RA liraglutide, but not saxagliptin (129), changed the overall structure of the gut microbiota, especially some bacteria related to glucolipid metabolism and intestinal inflammation (130, 131). For example, liraglutide treatment of diabetic male rats changed the gut microbiota, such as increasing SCFA-producing bacteria (*Bacteroides* and *Lachnospiraceae*) and probiotics (*Bifidobacterium*) (132). In addition, liraglutide treatment in wild-type mice and db/db mice significantly increased the abundance of intestinal *Akkermansia muciniphila* (130, 133, 134). In humans, liraglutide significantly increased the diversity and richness of the gut microbiota, especially Bacteroidetes, Proteobacteria, and *Bacilli* (135). However, a recent randomized controlled trial suggested that liraglutide and sitagliptin did not change the alpha or beta diversity of the gut microbiota, when they were used as add-on therapies with metformin or sulfonylureas (136). Additionally, a fixed combination of liraglutide and degludec for 6 months did not change the microbiome biodiversity or community among a group of very old T2D subjects (mean age 82 years) (137). The possible reason was that the combination of drugs masked the effect. In addition, liraglutide can activate the sympathetic nervous system of the gut (138). In conclusion, the GLP-1 analog liraglutide modulated the gut microbiota structure.

DPP-4 inhibitors could improve oral glucose intolerance and raise plasma GLP-1 concentrations. Additionally, they impacted on the composition and function of the gut microbiota. Vildagliptin monotherapy reduced the *Bacteroidetes* species in treatment-naive T2D patients, similar to acarbose (89). However, DPP-4 inhibitors [linagliptin (139) and sitagliptin (140)] increased the abundance of Bacteroidetes and succinate in mice. Moreover, vildagliptin mainly decreased *Oscillibacter* spp. and increased *Lactobacillus* spp. and propionate in Western diet-fed mice (141) and Zucker diabetic fatty rats (142). Similarly, DPP-4 inhibitor (PKF-275-055 or vildagliptin) treatment was reported to significantly decrease Firmicutes/Bacteroidetes ratios and increase butyrate-producing bacteria in diabetic and obese mice, similar to metformin (143, 144). Overall, treatment with a DPP-4 inhibitor moderately corrected the dysbiosis of the microbiota in obese and T2D mice.

### Effect of GLP-1 on the gut microbiota is involved in the inflammatory response

Disturbance of the gut microbiota can promote endotoxemia and insulin resistance. Increased Gram-negative *Enterobacteriaceae* and decreased acetic acid-producing bacteria (such as *Bifidobacteria*) associated with T2D resulted in increased LPS release and decreased acetic acid, respectively. Then, LPS from the gut lumen binds Toll-like receptor 4 (TLR4) to damage the intestinal barrier (145), and serum LPS moderately increases, which is an inflammatory state of prediabetes (146, 147). However, EECs increased the incretion of GLP-1 after sensing LPS as compensation (41). Similarly, inflammatory cytokine IL-6 (148) also acts on gut endocrine L cells to promote GLP-1 secretion. GLP-1 exerts a variety of physiological functions, such as promoting insulin synthesis and secretion, increasing satiety, and reducing food intake by binding to GLP-1R (149). GLP-1R is expressed in intestinal intraepithelial lymphocytes, and the GLP-1R agonist exendin-4 significantly inhibits inflammatory cytokines and macrophage infiltration (59). Many interventions that increase GLP-1 levels improve the intestinal inflammatory response (Tables 1 and 2). These findings suggested that the mechanism of GLP-1 action on gut microbiota involved inflammatory responses.

### Gut hormones affect the composition and function of gut microbiota

The gut microbiota is symbiotic with EECs. Distinct EEC subtypes are scattered among the epithelial cells of the gut mucosa and secrete different hormones. L cells that produce GLP-1 and PYY are distributed toward the distal intestine and are finally high in the colon (64, 150). These gut peptides can influence appetite, satiety, and food types. In return, alterations in gut microbiota could also affect eating behaviors (151, 152). In addition, gut peptides could regulate intestinal motility and intestinal permeability (153). Drosophila peptides have antimicrobial effects (154) and then regulate gut microbiota composition and abundance. Food peptides are multifunctional and can prevent gut dysbiosis (155). For example, a novel peptide, D3, increased the abundance of *Akkermansia muciniphila* and also suppressed appetite to improve DIO (156). Some milk-derived short peptides can enhance intestinal barrier function (157). In conclusion, gut peptides mediate the crosstalk between the gut microbiota and the host.

## INTERVENTIONS THAT MAY AFFECT GUT MICROBIOTA PROMOTE GUT PEPTIDE SECRETION, SUCH AS GLP-1

### Oral antidiabetic drugs promoted the GLP-1 secretion

In addition to dietary factors, nonantibiotic drugs also affect the microbiota composition and function. In turn, the gut microbiota can influence the effects of drugs. A well-known example is that Nature published an article providing support for the microbiota variation associated with the oral antidiabetic drug metformin in 2015. Treatment with metformin in T2D patients increased *Escherichia* spp. and decreased *Intestinibacter* spp. compared to untreated patients (158). In addition, a recent meta-analysis indicated that antidiabetic drugs (metformin) have a strong association with the relative abundance of microbiota (159).

In T2D patients, oral metformin increased the abundance of *Phascolarctobacterium*, *Intestinimonas,* and *Clostridium* III and the levels of GLP-1 and PYY (45, 88). However, stopping metformin decreased the Bacteroidetes abundance and the GLP-1 concentrations (87). Treatment with acarbose or vildagliptin in treatment-naive T2D patients decreased the abundance of Bacteroidetes and increased GLP-1 levels.

In DIO mice, the abundance of *Akkermansia muciniphila*, *Bacteroides*, *Butyricimonas*, and *Parabacteroides* was significantly increased by metformin treatment (107, 160). However, fecal transplantation from metformin-treated 16-week-old mice increased the GLP-1 concentration without changing the composition of gut microbiota and body weight (106). In hyperglycemic rats, SGLT2 inhibitor, canagliflozin, can also inhibit intestinal SGLT1, which is the primary transporter for glucose and galactose, to elevate plasma active GLP-1 level and reduce post-prandial glucose (108). Moreover, canagliflozin increased cecal SCFA production and changed the intestinal microbiota in renal failure mice (109). Dual SGLT1/2 inhibitors, sotagliflozin and licogliflozin, exert more selectivity for SGLT1 than canagliflozin, which may give dual SGLT1/2 inhibitors specific anti-hyperglycemia efficacy and cardiovascular and renal safety characteristics (161). So, although SGLT2 inhibitors are considered to act mainly through the kidneys, their effects on the microbiome deserve further evaluation. Therefore, the relationship between increased GLP-1 concentrations and gut microbiota after antidiabetic drug administration needs to be further confirmed.

### Bariatric surgery promoted the GLP-1 secretion

Bariatric surgery, which alters gut microbiota ecology, improved obesity and T2D well (11, 162). After Roux-en-Y gastric bypass (RYGB) or sleeve gastrectomy (SG) surgery, significant weight loss was exhibited along with the improved glycemia and changed gut microbiota. One of the main mechanisms is increased endogenous GLP-1 signaling (163). For example, RYGB surgery decreased Firmicutes and Archaea and increased Gammaproteobacteria (90). Therefore, in bariatric surgery, what is the exact relationship between gut microbiota and GLP-1 is still not clear.

Increased GLP-1 after bariatric surgery may be the result of rapid gastrointestinal nutrient input and increased plasma BAs (164). In healthy subjects, postprandial plasma BA concentrations were positively correlated with GLP-1 and PYY (165). In DIO mice, SG increased the LCA levels in portal veins without changing the abundance of *Clostridiales* to stimulate the GLP-1 production (11). Therefore, future studies of the crosstalk between gut microbiota and GLP-1 in bariatric surgery should start with BAs.

## CONCLUSION AND PROSPECTS

The interaction between GLP-1 and gut microbiota influences the host metabolism and health. Hosts in different metabolic states or with specific preferences will consume in different kinds and contents of the diet. Food can be derived into metabolites of the gut microbiota under the action of the gut, such as SCFAs. Some gut microbiota metabolites promote the secretion of GLP-1. GLP-1 exerts an influence on the brain, intestine, and pancreas to improve host metabolism. In addition, some interventions, such as prebiotics, probiotics, antidiabetic drugs, and bariatric surgery, changed the composition and function of the gut microbiota and then exerted benefits on the body, suggesting that gut microbiota is a target for diseases, such as obesity and T2D.

However, the relationship between gut microbiota, GLP-1 secretion, and the host still has many black boxes to uncover. In the future, the development of multiomics technology will help to interpret the relationship between GLP-1 and gut microbiota. In the clinical application of GLP-1RA, the effects of gut microbiota should be considered, and individualized programs should be given.
